# Alpha 2 Na^+^,K^+^-ATPase silencing induces loss of inflammatory response and ouabain protection in glial cells

**DOI:** 10.1038/s41598-017-05075-9

**Published:** 2017-07-07

**Authors:** Paula F. Kinoshita, Lidia M. Yshii, Ana Maria M. Orellana, Amanda G. Paixão, Andrea R. Vasconcelos, Larissa de Sá Lima, Elisa M. Kawamoto, Cristoforo Scavone

**Affiliations:** 1 Department of Pharmacology, Instituto de Ciências Biomédicas, Universidade de São Paulo, São Paulo, Brazil; 2 INSERM UMR U1043 - CNRS U5282, Université de Toulouse, UPS, Centre de Physiopathologie de Toulouse Purpan, Toulouse, 31300 France

## Abstract

Ouabain (OUA) is a cardiac glycoside that binds to Na^+^,K^+^-ATPase (NKA), a conserved membrane protein that controls cell transmembrane ionic concentrations and requires ATP hydrolysis. At nM concentrations, OUA activates signaling pathways that are not related to its typical inhibitory effect on the NKA pump. Activation of these signaling pathways protects against some types of injury of the kidneys and central nervous system. There are 4 isoforms of the alpha subunit of NKA, which are differentially distributed across tissues and may have different physiological roles. Glial cells are important regulators of injury and inflammation in the brain and express the α1 and α2 NKA isoforms. This study investigated the role of α2 NKA in OUA modulation of the neuroinflammatory response induced by lipopolysaccharide (LPS) in mouse primary glial cell cultures. LPS treatment increased lactate dehydrogenase release, while OUA did not decrease cell viability and blocked LPS-induced NF-κB activation. Silencing α2 NKA prevented ERK and NF-κB activation by LPS. α2 NKA also regulates TNF-α and IL-1β levels. The data reported here indicate a significant role of α2 NKA in regulating central LPS effects, with implications in the associated neuroinflammatory processes.

## Introduction

Na^+^,K^+^-ATPase (NKA) is an essential membrane protein due to its maintenance of cellular resting potential and osmotic balance^1^. NKA requires an ATP molecule to maintain high intracellular K^+^ concentrations and low Na^+^ concentrations, which are important for cellular function and neuronal transmission^2^.

Functional NKA has α and β subunits^3^. The α subunit is the catalytic subunit and the binding site of cardiotonic steroids, such as ouabain (OUA). All of the NKA subunits have different isoforms^4^. The α subunit has 4 isoforms, among which the α1 isoform is expressed in all cells^5^. The various isoforms have differing sensitivities to cardiotonic steroids. In mice, the α4 isoform is more sensitive to OUA than the other isoforms, while the α1 isoform is the least sensitive^6^.

Mutations in the α subunit have been recently reviewed^7^, indicating a role of such mutations in an array of medical conditions, including primary aldosteronism^8^, familial hemiplegic migraine (FHM)^9^, alternating hemiplegia of childhood (AHC)^10^, cerebellar ataxia, areflexia, pes cavus, optic atrophy, sensorineural hearing loss (CAPOS syndrome)^11^ and rapid-onset dystonia-parkinsonism (RDP)^12^.

Moreover, NKA has a non-pumping function *via* its action as a signal transducer^13^. Xie and Askari^14^ demonstrated that NKA activates the Src-Ras-MAPK pathway, which is involved in many cell processes such as growth, apoptosis and adhesion^15–17^. NKA also participates in inositol trisphosphate receptor (IP3R) activation, which evokes calcium oscillations by the release of Ca^2+^ from the endoplasmic reticulum^18^.

OUA is extracted from *Strophantus gratus* and is a hormone produced endogenously by the adrenal gland and the hypothalamus^19^. However, its physiological roles remain unclear. OUA inhibits NKA at high doses, thereby inducing an abnormal increase in intracellular Ca^+2^ and Na^+^, which triggers apoptosis. High doses of OUA in the central nervous system (CNS) can be used to develop models of mania^20^, while OUA can afford protection at low doses, as evidenced in kidney studies^21, 22^.

In a recent study^23^, OUA was shown to be protective against lipopolysaccharide (LPS) in the hippocampus, decreasing the LPS-induced increase in the mRNA levels of interleukin 1 beta (IL-1β), inducible nitric oxide synthase (iNOS), and the Bcl-2-associated X protein (Bax)/B-cell lymphoma 2 (Bcl-2) ratio, suggesting that OUA exerts anti-inflammatory and anti-apoptotic effects.

Further investigations of the roles of the different α NKA isoforms will help clarify the mechanisms of the protective effects of low-dose OUA. In the CNS, neurons express the α1 and α3 isoforms, while glial cells express the α1 and α2 isoforms, suggesting differences in the regulation of function and intracellular pathways of the isoforms among different cells in the CNS^24^.

Glial cells play important roles in the CNS, and they are no longer thought to simply provide neuronal support. In addition to recognizing and responding to damage stimuli, recent studies have shown that glial cells can regulate neuronal protection, repair and regeneration, control extracellular pH, and regulate action potential speed by modulating myelin production^25–28^.

Low doses of ammonia are toxic in the brain, including ammonia that arises from renal failure. In rat astrocyte cultures, low-dose ammonia increases the expression of α2 NKA and inhibits NKA activity^29^. In the CNS, only astrocytic NKA is stimulated by elevated K^+^^30^. Astrocytic NKA also controls glycolysis and mitochondrial activity due to the high rate of ATP consumption by brain NKA^31, 32^. Overall astrocytes play a powerful role in brain regulation, and astrocyte NKA contributes to the regulatory mechanism.

Neuroinflammation is a critical factor in neurodegenerative diseases, including Parkinson’s disease and Alzheimer’s disease. LPS-treated CNS cells can serve as a model of neuroinflammation. LPS is found in gram-negative bacteria and binds to *toll-like* receptor 4 (TLR4), which activates the transcription factor NF-κB in glial cells. NF-κB is an important transcriptional regulator of inflammatory cytokines and proteins. The present study investigated the role of glial α2 NKA during OUA modulation of the neuroinflammatory response induced by LPS.

## Results

### Glial cell responses to LPS and OUA

**Figure 1:**
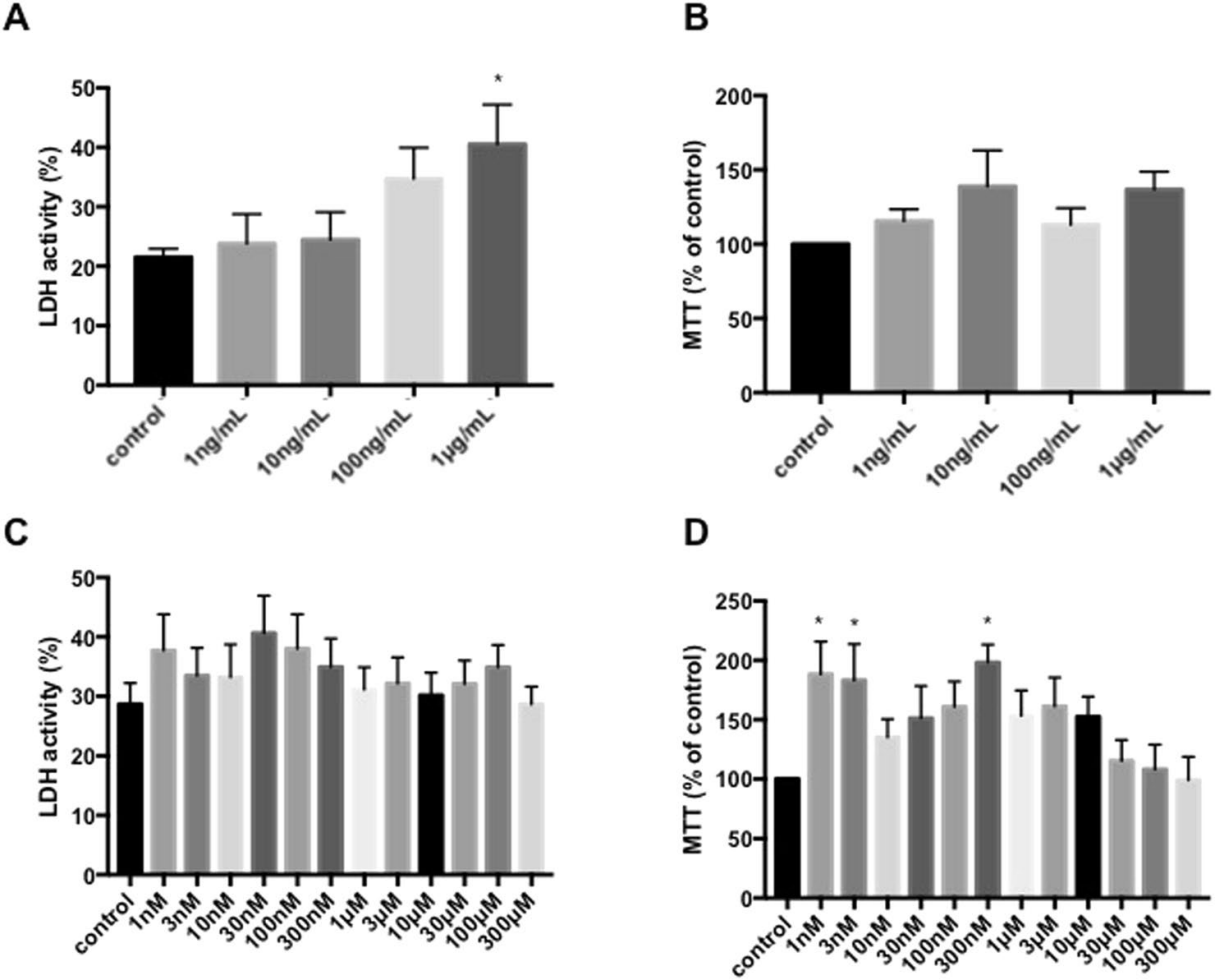
Effects of LPS and OUA in cell viability. (**A**) LDH activity of primary cultures of glial cells after 24 hours of LPS (1 ng/mL to 1 µg/mL) treatment (*p < 0.05). (**B**) MTT assay of the same cells used for LDH assay were incubated with MTT for 150 minutes, and the results were calculated by the percentage of each sample compared with the control group (n = 6). (**C**) LDH activity of primary cultures of glial cells after 24 hours of treatment with different OUA concentrations (1 nM-300 µM). (**D**) MTT assay of the same cells used for the LDH assay were incubated with MTT for 150 minutes, and the results were calculated by the percentage of each sample compared with the control group (n = 9) (*p < 0.05).

Cell viability was observed by LDH release and MTT assays. LPS treatment (1 µg/mL) increased LDH release and did not change the MTT response. This dose was used for further experiments. Surprisingly, glial cells were resistant to OUA-mediated cell death despite the fact that high concentrations of OUA inhibit NKA and cause excitotoxicity^33^. For a range of OUA concentrations (1 nM–300 µM), LDH release was not different after 24 hours of treatment compared with control, while the MTT response increased at some OUA concentrations (1 nM, 3 nM and 300 nM). However, both the MTT and LDH results indicated that OUA did not decrease cell viability (Fig. 1).

### OUA rescues NF-kB activation induced by LPS

**Figure 2:**
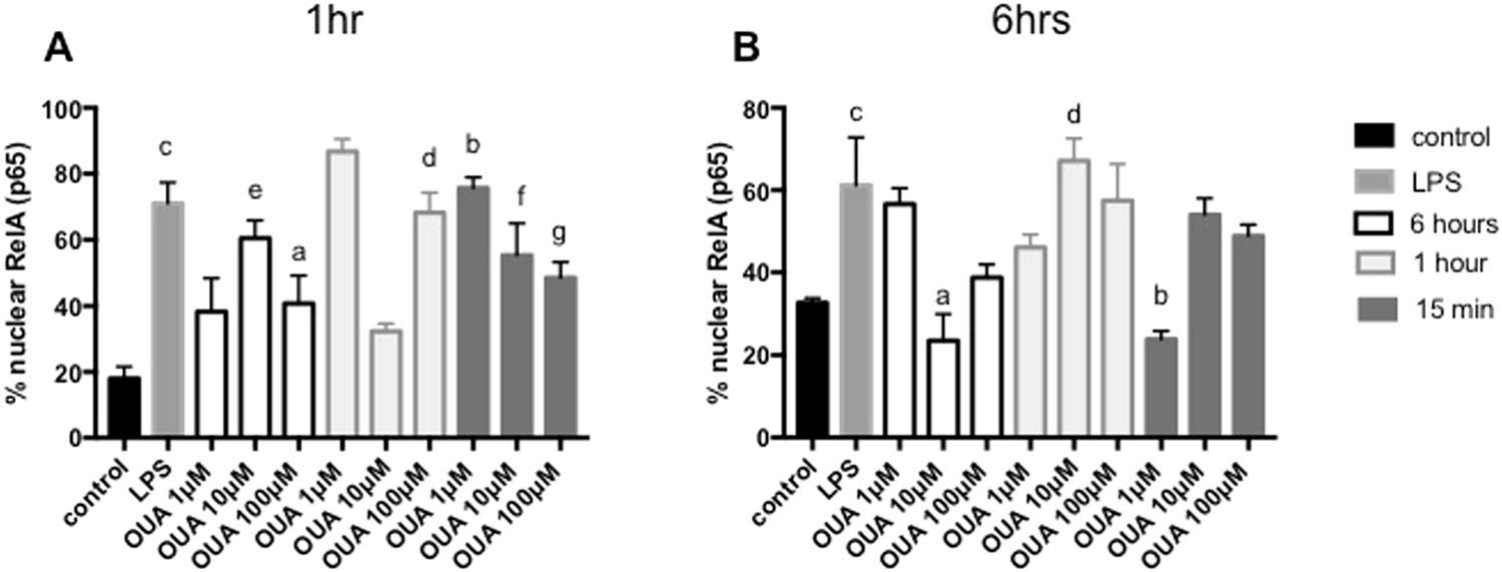
OUA decreases the nuclear translocation of RelA induced by LPS. (**A**) Glial cells were treated for 1 hour with LPS (1 µg/mL) followed by OUA treatment at different times (15 minutes,1 hour and 6 hours) and concentrations (1–100 µM) and were then stained with RelA antibody and DAPI. The results were calculated by the ratio of the number of cells with nuclear translocation of RelA to the total number of cells that were stained with DAPI (n = 4). a indicates p < 0.05 vs control, 10 µM OUA at 1 hour, 1 µM OUA at 6 hours, 100 µM OUA at 6 hours, 100 µM OUA at 15 minutes and 10 µM OUA at 15 minutes; b indicates p < 0.05 vs control, 10 µM OUA at 1 hour, 1 µM OUA at 6 hours and 100 µM OUA at 6 hours; c indicates p < 0.05 vs control, 10 µM OUA at 1 hour, 1 µM OUA at 6 hours and 1 µM OUA at 6 hours; d indicates p < 0.05 vs control, 10 µM OUA at 1 hour, 1 µM OUA at 6 hours and 1 µM OUA at 6 hours; and e, f, and g indicate p < 0.05 vs control. (**B**) Glial cells were treated for 6 hours with LPS followed by OUA treatment at different times (15 minutes, 1 hour and 6 hours) and concentrations (1–100 µM) and stained with RelA antibody and DAPI (n = 4). The results were calculated as the ratio of the number of cells with nuclear translocation of RelA to the total number of cells that were stained with DAPI. a indicates p < 0.05 vs 10 µM OUA at 1 hour, LPS, 100 µM OUA at 1 hour, 1 µM OUA at 6 hours and 10 µM OUA at 15 minutes; b indicates p < 0.05 vs control, 10 µM OUA at 1 hour, LPS, 100 µM OUA at 1 hour, 1 µM OUA at 6 hours and 10 µM OUA at 15 minutes; c and d indicate p < 0.05 vs control.

To identify the concentration and times that OUA protected against LPS activation, we performed immunofluorescence staining of RelA (p65), which is a NF-kB subunit that is activated during LPS-induced inflammation. Pre-treatment of glial cells with concentrations of 1, 10 and 100 µM OUA for 15 minutes, 1 hour and 6 hours, followed by challenge with LPS at 1 hour and 6 hours (Fig. 2) confirmed increased RelA nuclear translocation by LPS *vs* controls at both times.

**Figure 3:**
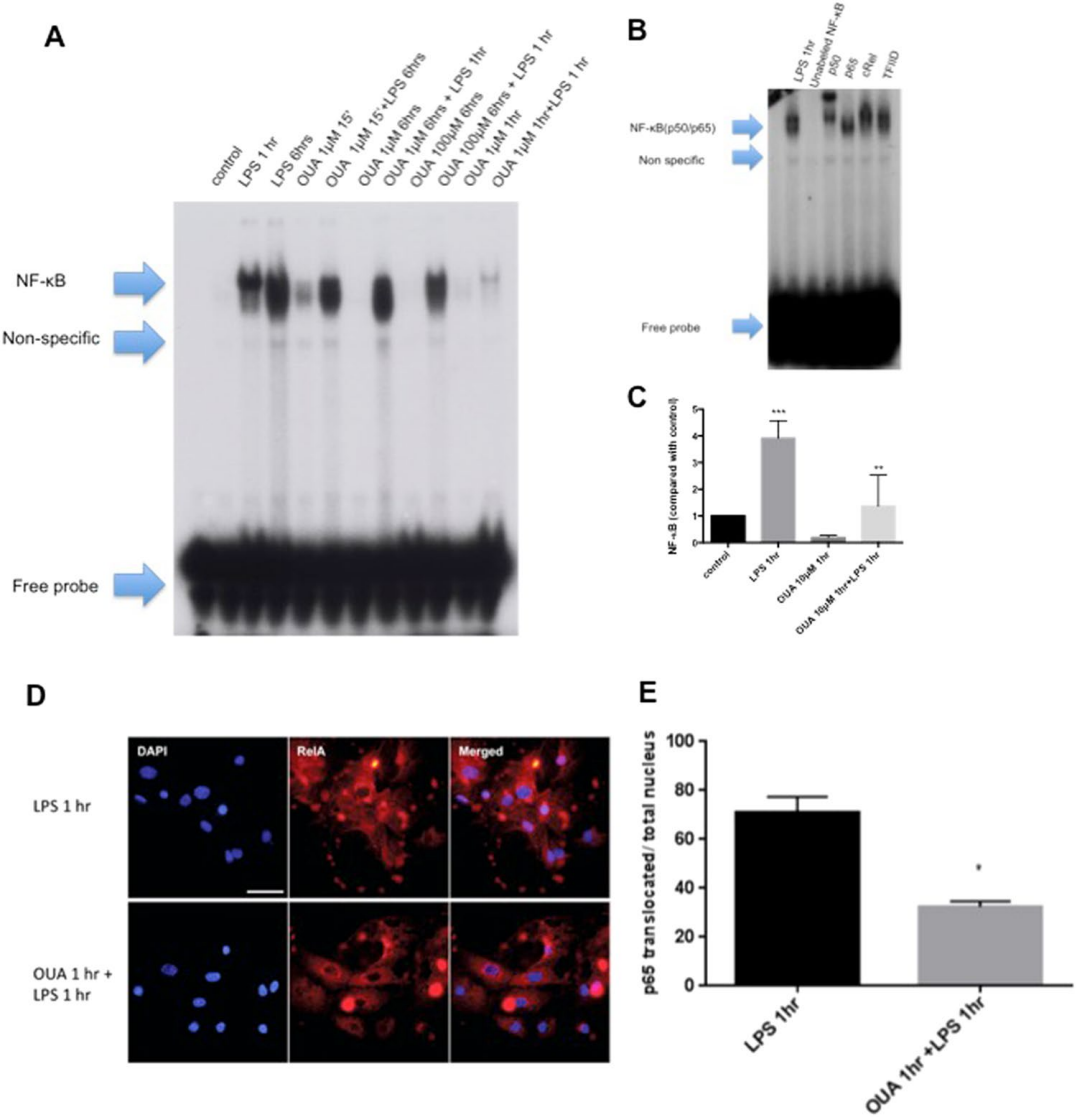
OUA can decrease the NF-κB activity induced by LPS in nuclear fraction. (**A**) After treatment with the given concentrations and time, protein from the cells was extracted, and the nuclear fraction was used to perform the EMSA assay to measure NF-kB activity. (**B**) A super-shift was also performed to show which NF-κB subunits are involved in this phenomenon. (**C**) Densitometric analysis comparing control NF-κB activity with 1 µg/mL LPS treatment for 1 hour and 10 µM OUA for 1 hour (n = 4). ***(p < 0.05 vs control and 10 µM OUA for 1 hour) and **10 μM OUA + LPS 1 hour (p < 0.05 vs LPS for 1 hour). (**D**) Glial cells were treated for 1 hour with LPS followed by 10 μM OUA treatment for 1 hour and 6 hours and stained with RelA antibody and DAPI. (**E**) The RelA (p65)-positive nuclei were counted and divided by the total number of nuclei, and the graph shows the comparison between the LPS group and for O + L group expressed by the ratio RelA (p65) translocated to the nucleus over the total amount of RelA (p65) (%) (n = 4).

RelA nuclear translocation induced by LPS was reverted at some OUA concentrations and times. Therefore, only the significant differences are shown in Fig. 2. These groups were submitted to an EMSA assay to more precisely detect whether the NF-kB that translocated to the nucleus was active and bound to its specific sequence in DNA. We compared the control group against LPS at 6 hours, 1 µM OUA treatment for 15 minutes, and 1 µM OUA treatment for 15 minutes + LPS at 6 hours (Fig. 3) groups.

While 1 µM OUA treatment for 15 minutes did not alter the basal activity of NF-kB, the pretreatment did not prevent LPS-induced NF-kB activation (Fig. 3). In the LPS at 1 hour groups, only 10 µM OUA treatment for 1 hour prevented NF-kB activation. A super-shift assay was also performed (Fig. 3) to clarify that NF-kB subunits are involved in this activation, especially regarding whether the RelA and p50 subunits are involved, which typically occurs following activation by LPS^34, 35^.

### Inflammatory response in α2-NKA and OUA protection

**Figure 4:**
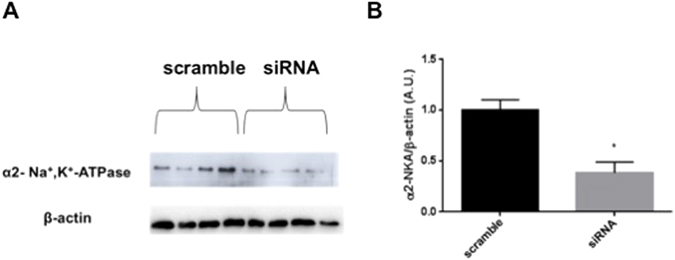
α2-Na^+^,K^+^-ATPase is silenced by siRNA. (**A**) Representative western blotting of glial cells after 72 hours of treatment with Lipofectamine RNAiMAX of α2-Na^+^,K^+^-ATPase siRNA and a scrambled sequence. (**B**) Western blotting bands were analyzed, and the α2/β-actin ratio was calculated (n = 6). *p < 0.05 *vs* scrambled sequence. The full-length blots are included in the Supplementary Information file.

After implementing the α2 NKA silencing protocol with siRNA, we performed a western blotting assay to validate α2 subunit silencing. A scrambled sequence did not alter the expression, whereas the isoform was silenced by α2 NKA siRNA (Fig. 4).

**Figure 5:**
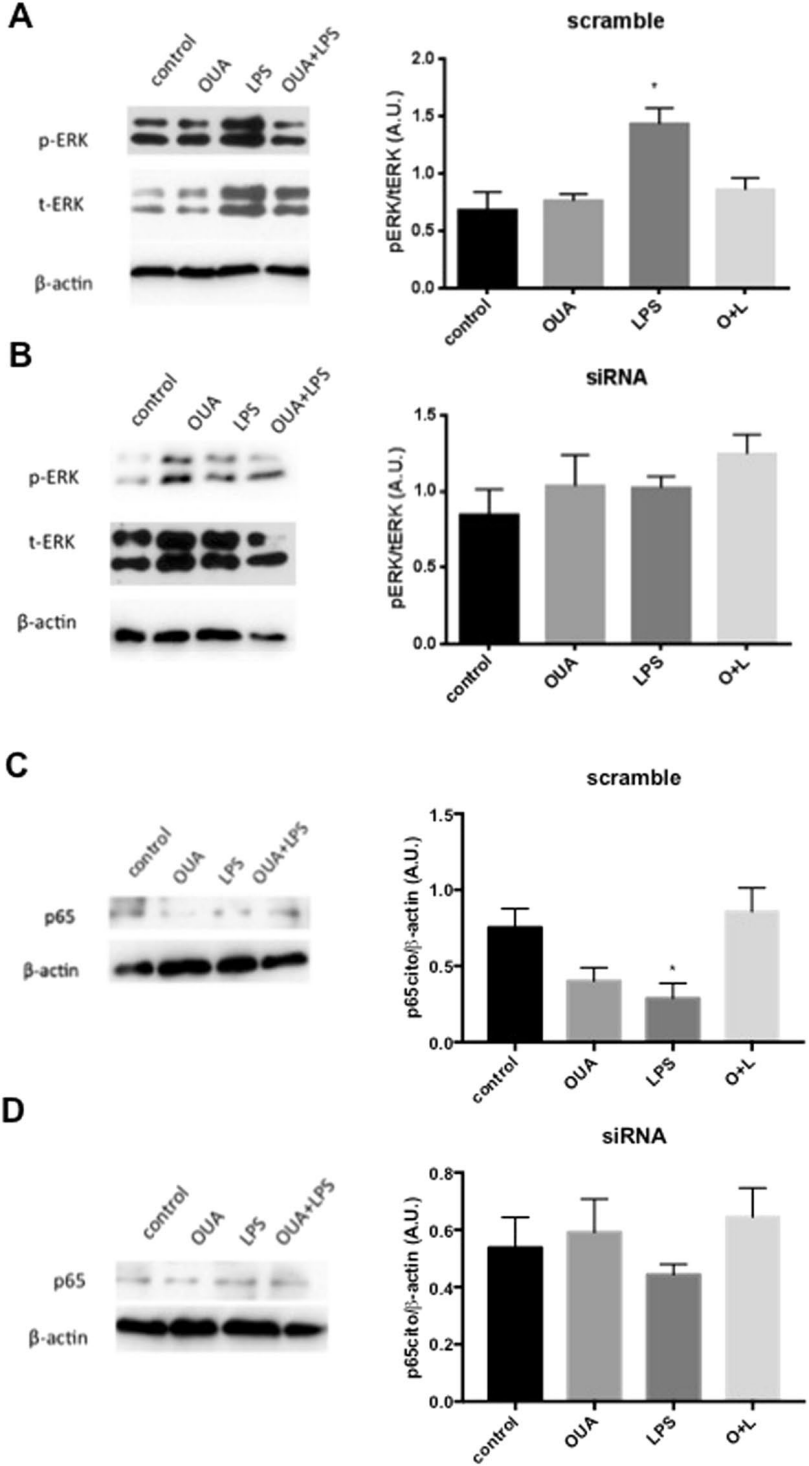
Anti-inflammatory effect of OUA against LPS and the effect of LPS in glial cells is lost after the silencing. The cytosolic fraction of protein from glial cells (siRNA or scramble) were extracted treated with OUA (10 µM) and LPS (1 µg/mL), and western blotting was performed for pERK, tERK, RelA (p65) and β-actin (n = 5). (**A**) Representative western blotting and densitometric analysis (arbitrary units, A.U.) of pERK/tERK ratio in the scramble group, *p < 0.05 *vs* control, OUA and O + L. (**B**) Representative western blotting and densitometric analysis (arbitrary units, A.U.) of the pERK/tERK ratio in the siRNA group. (**C**) Representative western blotting and densitometric analysis (arbitrary units, A.U.) of the RelA (p65)/β-actin ratio in the scrambled group, *p < 0.05 *vs* control and O + L. (**D**) Representative western blotting and densitometric analysis (arbitrary units, A.U.) of the RelA (p65)/β-actin ratio in the siRNA group. The full-length blots are included in the Supplementary Information file.

Furthermore, we tested the expression of phosphorylated *vs* total ERK (pERK/tERK), which is involved in LPS activation. LPS increased pERK, which was significantly different from all other groups (control, OUA and OUA + LPS) treated with the scrambled sequence. Pretreatment with OUA prevented the LPS-induced increase in pERK this group. However, this difference was not observed in the α2-silenced groups, as LPS did not activate ERK in these groups (Fig. 5).

To verify if OUA pretreatment was able to modulate the cytoplasmic levels of RelA, a western blotting assay of the cytoplasmic extract was performed. In the scramble group, LPS treatment decreased the expression of cytoplasmic RelA, as expected, indicating that LPS increased the nuclear translocation of RelA. Pretreatment with OUA followed by LPS also decreased RelA nuclear translocation, indicating an OUA-protective effect based on its anti-inflammatory action, as previously shown in the EMSA assay for NF-kB activity. However, the same pattern was not observed in the siRNA group, in which LPS did not decrease cytoplasmic RelA but exhibited similar levels to the other siRNA groups (Fig. 5).

**Figure 6:**
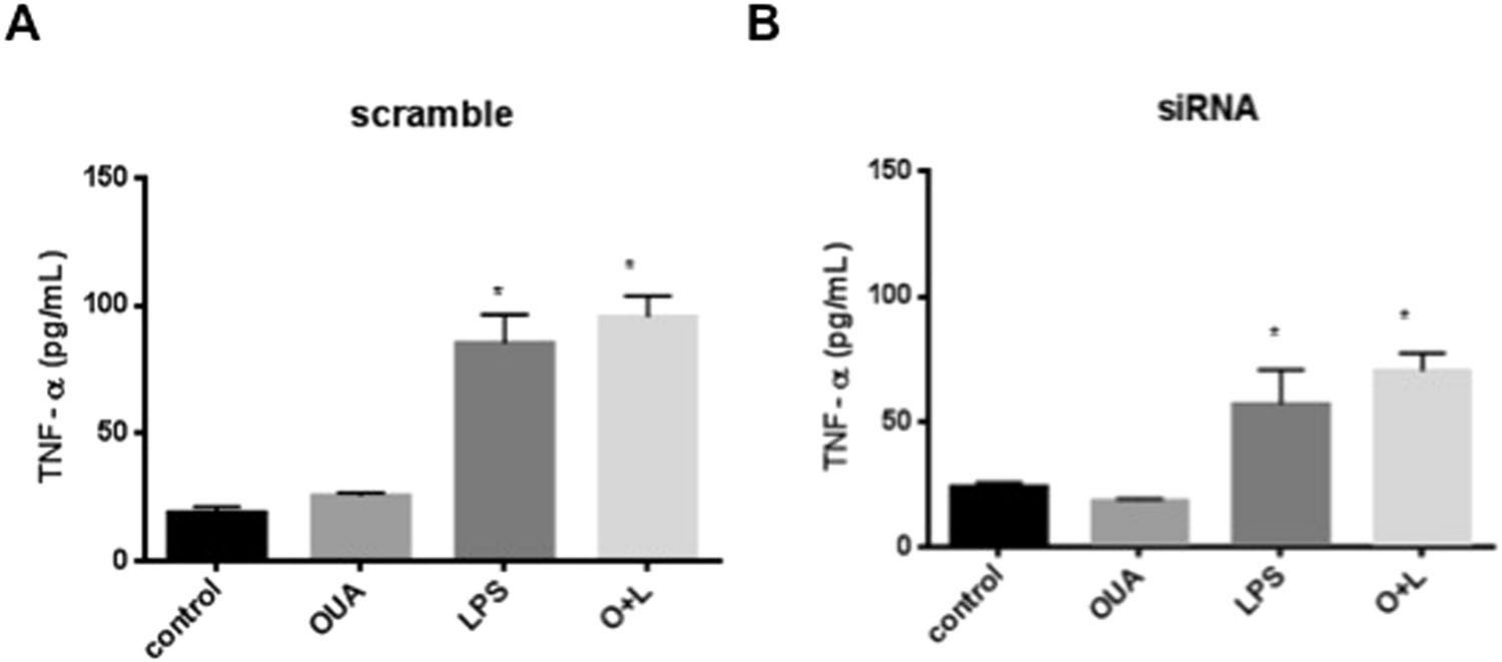
The lack of α2-Na^+^,K^+^-ATPase decreases TNF-α levels. Cell culture supernatants of scrambled sequence and siRNA cells after treatment with LPS (1 µg/mL) and OUA (10 µM) were collected and used to measure TNF-α levels by ELISA (n = 6). (**A**) The levels of TNF-α in the scrambled group, *p < 0.05 *vs* control and OUA. (**B**) The levels of TNF-α in the siRNA group, *p < 0.05 *vs* control and OUA. (**C**) Comparison of the levels of TNF-α in the siRNA and scramble groups, *p < 0.05 *vs* scramble + LPS and scramble + O + L.

**Figure 7:**
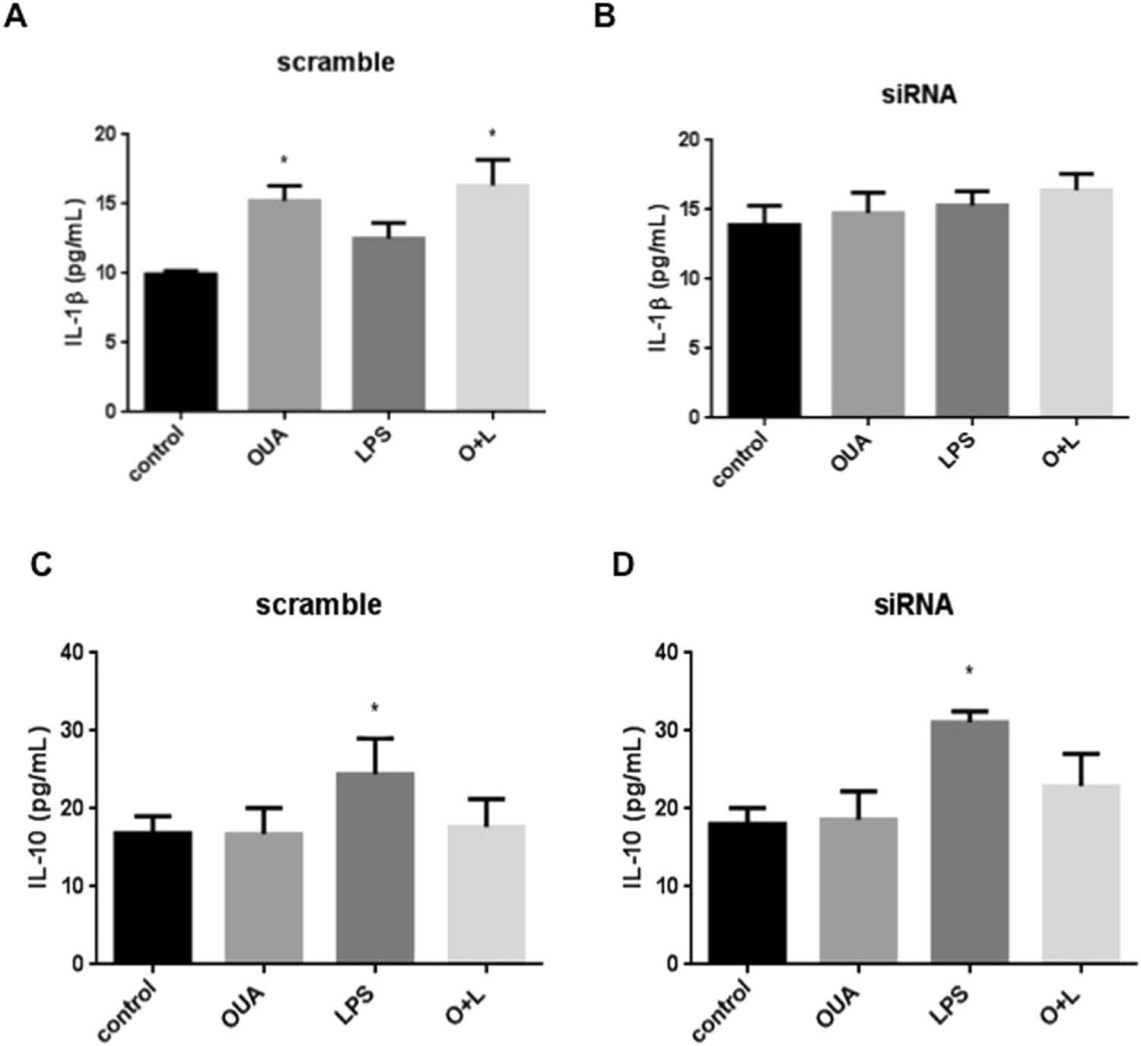
α2-Na^+^,K^+^-ATPase is not related to changes in IL-10 levels. Cell culture supernatants of scrambled sequence and siRNA cells after treatment with LPS(1 µg/mL) and OUA (10 µM) were collected and used to measure IL-1β and IL-10 levels by ELISA (n = 6). (**A**) The levels of IL-1β in the scrambled group, *p < 0.05 *vs* control. (**B**) The levels of IL-1β in the siRNA group showed no difference between the groups. (**C**) The levels of IL-10 in the scramble group, *p < 0.05 *vs* control, OUA and O + L. (**D**) The levels of IL-10 in the siRNA group, *p < 0.05 *vs* control, OUA and O + L.

TNF-α (Fig. 6) and IL-1β (Fig. 7) were measured in the supernatants of treated cells using an ELISA kit. TNF was increased in the LPS group, and OUA was not reduced in the scramble nor siRNA groups. However, the siRNA + LPS group TNF was less activated than the scramble + LPS and OUA + LPS groups, indicating that the LPS response was disrupted in the absence of the α2 isoform.

Although LPS did not increase IL-1β release in the scramble group, OUA and OUA + LPS increased IL-1β release, which was not observed in the siRNA group. Thus, the inflammatory response may be compromised in siRNA glial cells lacking the α2 isoform. IL-10 levels (Fig. 7) were increased in the LPS group compared to the scrambled and siRNA groups, indicating that the anti-inflammatory pathway is not compromised by the lack of the α2 isoform.

## Discussion

Glial cells play an important role in inflammatory responses, with microglia regarded as brain macrophages in part due to the similarity of their responses and their participation in CNS adaptive and innate immune responses^36^. Astrocytes play a pivotal role in neuroinflammation^37^ and neuronal loss^38^, which are common characteristics of neurodegenerative diseases.

Astrocytes can express TLR4, although their immune system regulatory role still requires clarification^39–42^. Cytokine release by microglia also activates astrocytes, in turn leading to astrocyte release of cytokines, including TNF-α and IL-1β^43^. In addition to regulating neuronal synapses, astrocytes are an integral part of the blood-brain barrier (BBB), and they regulate BBB permeability to infiltrating leukocytes, such as monocytes and lymphocytes. Such crucial coordinating roles highlight the importance of changes in astrocytes over the course of neuroinflammation^44, 45^.

Although the roles of astrocytes in neuroinflammation have been described in several studies, little is known regarding the role of the α2 isoform in this process. Neurons and astrocytes express the NKA isoforms differently. Therefore, because these cells have such different functions, the isoforms could also have highly distinct and specific roles. This is a pioneering study presenting the relationship of between the α2 isoform and inflammation in astrocytes and reveals that the α2 isoform exerts some control over inflammatory responses. This study also shows the relevance of the ouabain-NKA complex against inflammation triggered by LPS.

Glial NF-κB activation is inducible and is important in the regulation of many inflammatory process, with relevance to a host of pathologies including ischemia and Alzheimer’s disease^46^. In addition, classical NF-κB activation by LPS or pro-inflammatory cytokines, such as TNF-α, occurs *via* activation of IKK, which phosphorylates IκB and leads to the release of p50/RelA heterodimers. RelA is a NF-κB subunit that carries a transcription activating domain; therefore, RelA can initiate NF-κB-dependent genes in the nucleus. This heterodimer can be used as an inflammatory marker to study the influence of OUA on the regulation of LPS effects^46^.

The effect of OUA can also differ depending on cell type. Our group showed that OUA in cerebellar neurons can activate NF-κB *via* the NMDAr-Ras-Raf-ERK signaling cascade. This signaling cascade leads to increased *Bdnf* mRNA levels, indicating an important adaptive response^47^. However, OUA did not activate NF-κB in glial cells at the times measured in the present study, but rather decreased LPS-induced NF-κB activation. This finding indicates differential effects of OUA in glial and neuronal cells. Although different pathways may act on in these cells, OUA exhibited an anti-inflammatory effect that was previously observed in the hippocampus^23^. However, in this study, NF-κB activation did not occur upon treatment with 10 µM OUA, which could be due to differences in the time course, as NF-κB is cyclically activated^48^, or to differences in the cell type and/or OUA concentration tested. Higher concentrations of OUA may also activate NF-κB, as described by a previous study in which hippocampal astrocyte primary cultures treated with a high concentration (100 µM) of OUA exhibited increased NF-κB nuclear translocation based on activation of calcium oscillations^49^.

Although some studies have shown that 10 µM OUA can inhibit the α2 NKA isoform^50, 51^, this concentration did not induce significant apoptosis in glial cells, as shown by MTT and LDH assays, compared to the control group, as shown in Fig. 1, suggesting that no inhibition of NKA occurs. Furthermore, no association between NKA inhibition and the effects triggered by OUA was found in a previous work that used the same concentration in cerebellum primary cultures^52^. Dvela *et al*. demonstrated that OUA is present in fetal bovine serum (FBS) at the range of 0.3-0.4 nM^53^. As such, cells may already be adapted to the presence of nanomolar OUA concentrations, thereby preventing any noticeable effects if nanomolar OUA concentrations are applied.

The anti-inflammatory role of OUA has been investigated by a variety of approaches. Low doses of OUA can prevent LPS-induced changes in astrocytes, including IL-1β release and NKA down-regulation^54^. OUA decreased the inflammatory response in rats treated with carrageenan and zymosan, which were used to evoke paw edema. This inflammatory effect arises from inhibition of prostaglandin E2, bradykinin and mast cell degranulation^55^. Other anti-inflammatory effects involved include decreasing IL-6 and TNF-α release^56^ and blocking the TNF-α/NF-κB pathway, as has been demonstrated for other cardiotonic glycosides^57^.

OUA protection was also observed in an excitotoxicity model using kainic acid in the striatum, in which OUA increased the anti-apoptotic protein, Bcl-2^58^, as also supported by later findings^23^. Serum deprivation in embryonic kidney cells increases cell apoptosis, which was prevented by OUA *via* NF-κB modulation^21^. The same group also reported the same protective effect of OUA against Shiga toxin in kidney cells via increasing Bcl-2 expression and NF-κB activation^22^.

The differential expression, activity and regulation of the NKA isoforms indicate their distinct physiological roles. Pierre *et al*. showed that, in cells with different α isoforms co-expressed with the β1 isoform, only the α2 isoform failed to activate ERK after OUA application. This suggests that variations in the expression of α and β isoforms may differentially modulate second messenger signaling pathways in different cell types^59^.

In the CNS, the α2 isoform exerts significant effects, and mutations in this isoform are related to FHM^9^. The lack of one α2 isoform allele in mice increases blood pressure due to high sodium concentrations in the cerebrospinal fluid that arise from alterations in the central renin-angiotensin system^60^. α2 heterozygous mice exhibit increased anxiety, decreased locomotor activity and impaired spatial memory^61^. It was also shown that the α2 isoform may be more relevant than the α1 isoform in intracellular sodium concentration restoration after an increase in glutamate levels^62^.

Variations in the α2 isoform are also related to damage in the CNS, with kainic acid increasing α2 mRNA levels in the rat hippocampus^63^, which similarly increases when astrocytes are treated with ammonia^29^. As such, alterations in the α2 isoform can have significant consequences, both centrally and systemically.

LPS treatment in astrocytes activates ERK, a kinase activated in response to cellular stress^64^ that can also activate NF-κB^65^. LPS treatment also decreases cytoplasmic RelA levels, which occurs when NF-κB is translocated to the nucleus^66^. When the α2 isoform is silenced, the effect of LPS on ERK and RelA is decreased, indicating that this isoform regulates the response of glial cells to LPS perhaps *via* modulation of the TLR4 pathway. However, given the attenuated rise in TNF-α when the α2 isoform is silenced, it is likely that NF-κB activation is not the only pathway involved in TNF-α release.

Interestingly, treatment with LPS increased the release of IL-10, which was diminished by OUA in the scramble and siRNA groups, again indicating that OUA can antagonize the effects of LPS. However, this OUA effect was not dependent on the α2 isoform, which suggests that the protection effect is a result of the interaction of OUA with the α1 isoform.

The alterations in the TNF-α response to LPS in the scrambled group *vs* the α2 silenced group provide further support of the role of this isoform in the regulation of the inflammatory response. Other pro-inflammatory cytokine changes were also evident: LPS did not activate IL-1β, while OUA increased IL-1β release, which was lost when the α2 isoform was silenced. These findings corroborate previous studies that showed that OUA can upregulate *IL-1β* mRNA levels in the rat hippocampus^47^.

Thus, OUA may act to increase activation of the NLRP3 inflammasome, as suggested by previous work on NKA inhibition^67^. This would further suggest that IL-18 is increased, which is a significant inducer of the interferon-gamma. As IL-1β, IL-18 and especially interferon-gamma can all increase indoleamine 2,3-dioxygenase (IDO)^68^, the roles of IDO-driven tryptophan catabolites, such as the neuroprotective kynurenic acid and the excitotoxic quinolinic acid, on the effects of OUA in glia and the CNS require further investigations.

Another report in the literature supports the idea of a role of the α2 isoform in neuronal degeneration, which can also occur during neuroinflammatory processes. In a mutant superoxide dismutase 1 (SOD1) model of amyotrophic lateral sclerosis, expression of the astrocytic α2 isoform was increased, and knockdown of the astrocyte α2 isoform protected motor neurons from degeneration^69^. The authors used digoxin to inhibit the α2 isoform, which afforded protection, highlighting the relationship of this isoform with neurodegeneration. As such, the α2 isoform may be an important target for the treatment of neurodegenerative diseases^69^.

In primary cultures of murine cortex glial cells, pretreatment with 10 µM OUA prevented NF-κB activation, as driven by inflammatory stimuli, such as LPS, thereby reinforcing the previously reported anti-inflammatory effect of OUA. Notably, the data presented here show for the first time that α2 NKA isoform silencing decreases LPS-induced ERK activation and TNF-α release and modulates RelA nuclear translocation and, therefore, NF-κB activity. Overall, regulation of the astrocyte α2 isoform is a significant determinant of the nature of inflammatory responses in the brain, with consequences in an array of neurodegenerative conditions.

## Materials and Methods

### Chemicals

Cell culture reagents were purchased from Thermo Fisher Scientific. A protein assay kit was purchased from Bio-Rad. Routine reagents, LPS from *Escherichia* c*oli* O111:B4 and OUA were purchased from Sigma Chemicals. All solutions were prepared immediately before use.

### Animal conditions and ethics

All experiments were performed *in vitro* after dissection of cortex from C57BL6 mouse neonates. The animals used for breeding were housed under a 12-hour light-dark cycle (lights on at 7 am) and had free access to water and food. All animal procedures were in accordance with The Ethical Principle in Animal Research adopted by the Brazilian College of Animal Experimentation (COBEA) and were approved by the Ethical Committee for Animal Research (CEEA) of the Biomedical Sciences Institute of the Universidade de São Paulo, São Paulo, São Paulo State, Brazil. The protocol was registered under number 37 on page 101 of book 02 of animals used for experimentation^70^.

### Preparation of primary cortex glial cell culture

Primary cortex glial cells were prepared from postnatal (P1-P4) mice. After euthanasia, the meninges were separated from the brain, and the cortex was dissected in cold Hank’s Balanced Salt Solution (HBSS). The tissue was dissociated with trypsin (0.25%) at 37 °C for 5 minutes. DMEM (glutamine 4 mM, 10% FBS and 1% penicillin/streptomycin) was added to the solution to stop digestion, and cells were dissociated with a Pasteur pipette. The cell pellet was filtered using a cell strainer. Cells were then counted in a Neubauer chamber, and each Flask T75 was plated with 1 × 10^6^ cells (modified from 70). Cells were kept in a SL Shel Lab (CO_2_ series, Sheldon Mfg Inc.) incubator at 37 °C with 5% CO_2_. The medium was changed every 3 days, and the cultures were maintained for 10–14 days.

Cells were plated in 6-well or 24-well plates for experiments. The cells were trypsinized (0.25%) at 37 °C for 10 minutes to detach the cells from the flask. The reaction was stopped with DMEM, and the cells were transferred to a tube and centrifuged for 2 minutes at 2000 rpm. The supernatant was discarded, and the pellet was resuspended in DMEM and counted for plating. Each well was plated with 1 × 10^6^ cells in 6-well plates and plated with 1 × 10^4^ cells for 24-well plates. The culture used for the experiments was considered an astroglial-enriched culture because there were 91.2% astrocytes labeled with GFAP.

### Determination of cell viability by LDH release and MTT assay

Cell viability was estimated by a Cytotox 96 non-radioactive assay (Promega). The assay was performed according to the manufacturer’s instructions. 1 × 10^4^ cells per well were plated in 24-well plates. Lactate dehydrogenase (LDH) release was assayed after 24 or 48 hours of treatment with OUA or LPS by removing 50 μl supernatant from each well and incubating with Cytotox 96 reagent for 30 minutes covered with aluminum foil at room temperature. Stop solution was added, and the absorbance was measured at 490 nm. The percentage of LDH activity was measured by the ratio (absorbance of the sample/absorbance of maximum activity) × 100.

After the LDH activity assay, cells were incubated with filtered MTT solution in DMEM without BSA (0.05 mg/mL) at 37 °C for 150 minutes. The supernatant was removed from the plate, and DMSO was added. The new supernatant was plated in another plate and measured at 570 nm. MTT% relative to controls was calculated according to the ratio of the absorbances of the sample and the control, and both were corrected by the absorbance of DMSO (blank) for each experiment. Control cells did not receive any LPS or OUA treatment.

MTT (%) = (absorbance of the sample − absorbance of DMSO)/(absorbance of the control − absorbance of DMSO) × 100.

### Treatment

The dose-response of OUA and LPS treatments was assessed for different concentrations (OUA 1 nM - 300 μM and LPS 1 ng/mL-1 μg/mL) and time points (24 and 48 hours) to observe cell viability. After this first screening, only OUA concentrations from 1 μM-100 μM and LPS 1 μg/mL were tested at different time points (OUA at 15 minutes, 1 hour and 6 hours; LPS at 1 hour and 6 hours). The concentrations chosen for western blotting and ELISA were 10 μM OUA and 1 μg/mL LPS both for 1 hour treatment.

### Immunofluorescence

After treatment, the media was removed from the plate, and the coverslips were washed twice with cold PBS. Cells were fixed with methanol for 10 minutes at room temperature and washed with PBS three times for 5 minutes. The fixed cells were incubated with blocking serum (5% normal donkey serum in Triton X-100, 0.01%) for one hour and incubated overnight with primary antibodies (p65, Santa Cruz Biotechnology, 1:200). The primary antibody was removed, and the plate was washed with blocking serum three times for 10 minutes. The cells were incubated with secondary antibody (Alexa 594 donkey anti-rabbit, Invitrogen, 1:1000) and diluted in PBS with 0.01% Triton X-100 for 2 hours while protected from light. The coverslips were washed five times with PBS for 5 minutes, incubated with DAPI (1:100,000), and washed twice. Then, the coverslips were placed in a microscope glass with mounting media solution and analyzed under a Nikon Eclipse 80i fluorescence microscope with a DXM 1200 C digital camera (Nikon). The objective used was a Plan Fluor 20× 0.5 Dic M/N2 from Nikon (Japan).

### Protein extraction from the nucleus and cytoplasm

Culture media were removed, and the cells were scraped in cold PBS with 0.5 mM PMSF and centrifuged at 4 °C for 2 minutes at 13,000 × *g*. The pellet was resuspended in lysis buffer (10 mM HEPES, pH 7.9, 10 mM KCl, 1.5 mM MgCl_2_, 0.5 mM PMSF, 0.1 mM EDTA, 2 μg/mL leupeptin, 2 μg/mL antipain, 30 mM NaF, 3 mM sodium orthovanadate and 20 mM sodium pyrophosphate) and incubated on ice for 15 minutes. NP-40 was added, and the samples were homogenized and centrifuged for 30 s at 13,000 × *g* at 4 °C. The supernatants were used for western blotting assay, and the pellets were resuspended in extraction buffer (1.5 mM MgCl_2_, 20 mM HEPES, pH 7.9, 25% glycerol, 300 mM NaCl, 0.5 mM PMSF, 0.25 mM EDTA, 2 μg/mL leupeptin, 2 μg/mL antipain, 3 mM sodium orthovanadate, 30 mM NaF and 20 mM sodium pyrophosphate) and kept on ice for 20 minutes. Samples were centrifuged for 20 minutes at 13,000 × *g* at 4 °C, and the supernatants were aliquoted as the nuclear extract used for EMSA assay. Protein concentration was determined using Bio-Rad protein reagent.

### EMSA

Double-stranded oligonucleotides containing the NF-κB consensus sequence (5′-AGTTGAGGGGACTTTCCCAGGC-3′) from Promega were end labeled in the presence of radioactive γ-32P dATP using T4 polynucleotide kinase (Promega). Nuclear extracts (2.5 μg) were incubated with the 32P-labeled probe. Then, the nuclear extracts are incubated for 30 minutes at room temperature with a reaction buffer (250 mM NaCl, 50 mM Tris-HCl pH 7.5, 5 mM MgCl_2_, 20% glycerol, 2.5 mM EDTA, 0.25 μg/μL of poly(dI-dC) and 2.5 mM dithiothreitol). Sample-NF-κB probe complexes were separated by electrophoresis through a 6% acrylamide:bis-acrylamide (37.5:1) gel in TBE (45 mM boric acid, 45 mM Tris, 0.5 mM EDTA) for 2 hours at 150 V. Gels were vacuum dried for 1 hours at 80 °C and exposed to X-ray film at −80 °C.

In competition assays, 2.5 μg of nuclear extract was incubated with a specific competitor (unlabeled double-stranded NF-κB consensus oligonucleotide) and a non-specific competitor (unlabeled transcription initiation factor IID [TFIID]). For supershift assay, antibodies against subunits of NF-κB (p50, p65, cRel) (Santa Cruz Biotechnology) were added to the binding reactions. Autoradiographs were visualized using a photodocumentation system DP-001-FDC and quantified with ImageJ (NIH) software^70^.

### α2 silencing with siRNA

After 11 to 14 days of culture glial cells were trypsin dissociated and 0.7 × 10^6^ cells were plated in each well in a 6-well plate. When the culture reached 70% of confluence, siRNA-lipid complexes were prepared with RNAiMAX (Thermo Fisher Scientific) by following the manufacturer’s protocol. The Na^+^/K^+^-ATPase α2 siRNA (Santa Cruz Biotechnology) was used for the experiments and the scramble sequence used was Negative Control High GC Duplex (Invitrogen). After 6 hours, fresh DMEM medium was added, being completely changed after 24 hours with the treatment with OUA (10 µM) and LPS (1 µg/mL) being done the following day^71^.

### Western blotting

Electrophoresis was performed using 10% polyacrylamide gel and a Bio-Rad mini-Protean III apparatus. In brief, proteins present in the cytosolic fraction were size-separated via 10% SDS-PAGE (90 V). The proteins were blotted onto nitrocellulose membranes (Bio-Rad, Hercules, CA, USA) and incubated with the specific RelA (p65) (sc-0372; Santa Cruz Biotechnology, Santa Cruz, CA, USA), α2 Na^+^,K^+^-ATPase (Millipore), pERK (Cell Signaling) and t-ERK (Cell Signaling) antibodies followed by the secondary antibody (rabbit). Proteins recognized by the antibodies were revealed via an electrochemiluminescence (ECL) technique according to the manufacturer’s instructions (Amersham Biosciences, Amersham, UK). To standardize and quantify the immunoblots, we used a photodocumentation system (DP-001-FDC) and ImageJ (NIH) software. Several exposure times were analyzed to ensure the linearity of the band intensities. β-actin antibody (sc-1616; Santa Cruz Biotechnology, Santa Cruz, CA, USA) was used as an internal experimental control, and the results are expressed in relation to β-actin (Sigma) intensity^23^.

### ELISA kits

TNF-α, IL-10 and IL-1β immunoassay platinum kits were purchased from eBioscience. The kits were utilized according to manufacturer’s instructions, and the cell culture supernatant was used to measure the release of these cytokines. Briefly, samples were added to coated microwells with an antibody against TNF-α, IL-10 or IL-1β together with a biotin-conjugated antibody. Thus, the biotin-conjugated antibody binds to the cytokine bound to the coated antibody. The plate was incubated for 2 hours at 400 revolutions per minute (rpm) at room temperature. The wells were washed, and streptavidin-HRP was added to the entire plate and maintained for 1 hour at 400 rpm in room temperature. After the wells were washed and then incubated with TMB Substrate Solution for 30 minutes while protected from light. The color was monitored, and the reaction was stopped with a stop solution. The absorbance was measured using a spectrophotometer at 450 nm. The concentrations of the cytokines were measured by correlating with the standard curve.

### Statistical analysis

The results are expressed as the mean ± SEM of the indicated number of experiments. Statistical comparisons for OUA-induced changes in mRNA levels, protein expression, ELISA and NKA activity were performed by one-way analysis of variance (ANOVA), followed by the Newman-Keuls post-test.

Unpaired *t*-tests with Welch’s correction were used to compare the expression of α2 isoforms against siRNA and scrambled sequences. All analyses were performed using the Prism 5 software package. *P*-values < 0.05 were considered to reflect statistically significant differences.

## Electronic supplementary material

Supplementary information
