# Supplementary material for: Alpha 2 Na+,K+-ATPase silencing induces loss of inflammatory response and ouabain protection in glial cells
Source: Sci Rep. 2017 Jul 7;7:4894. doi: 10.1038/s41598-017-05075-9 (PMC5501845; doi:10.1038/s41598-017-05075-9)
Supplement: Supplementary file 1 — Supplementary information [file 41598_2017_5075_MOESM1_ESM.pdf]

Alpha 2 Na+,K+-ATPase silencing induces loss of inflammatory response and ouabain protection in glial cells

Paula F. Kinoshita[1], Lidia M. Yshii[1,2], Ana Maria M. Orellana[1], Amanda G. Paixão[1], Andrea R. Vasconcelos[1], Larissa de Sá Lima[1], Elisa M. Kawamoto[1], and Cristoforo Scavone*[1]

[1]Department of Pharmacology, Instituto de Ciências Biomédicas, Universidade de São Paulo
[2]INSERM UMR U1043 - CNRS U5282, Université de Toulouse, UPS, Centre de Physiopathologie de Toulouse Purpan, Toulouse, 31300, France
*Corresponding author: Prof. Dr. Cristoforo Scavone, **criscavone@usp.br**

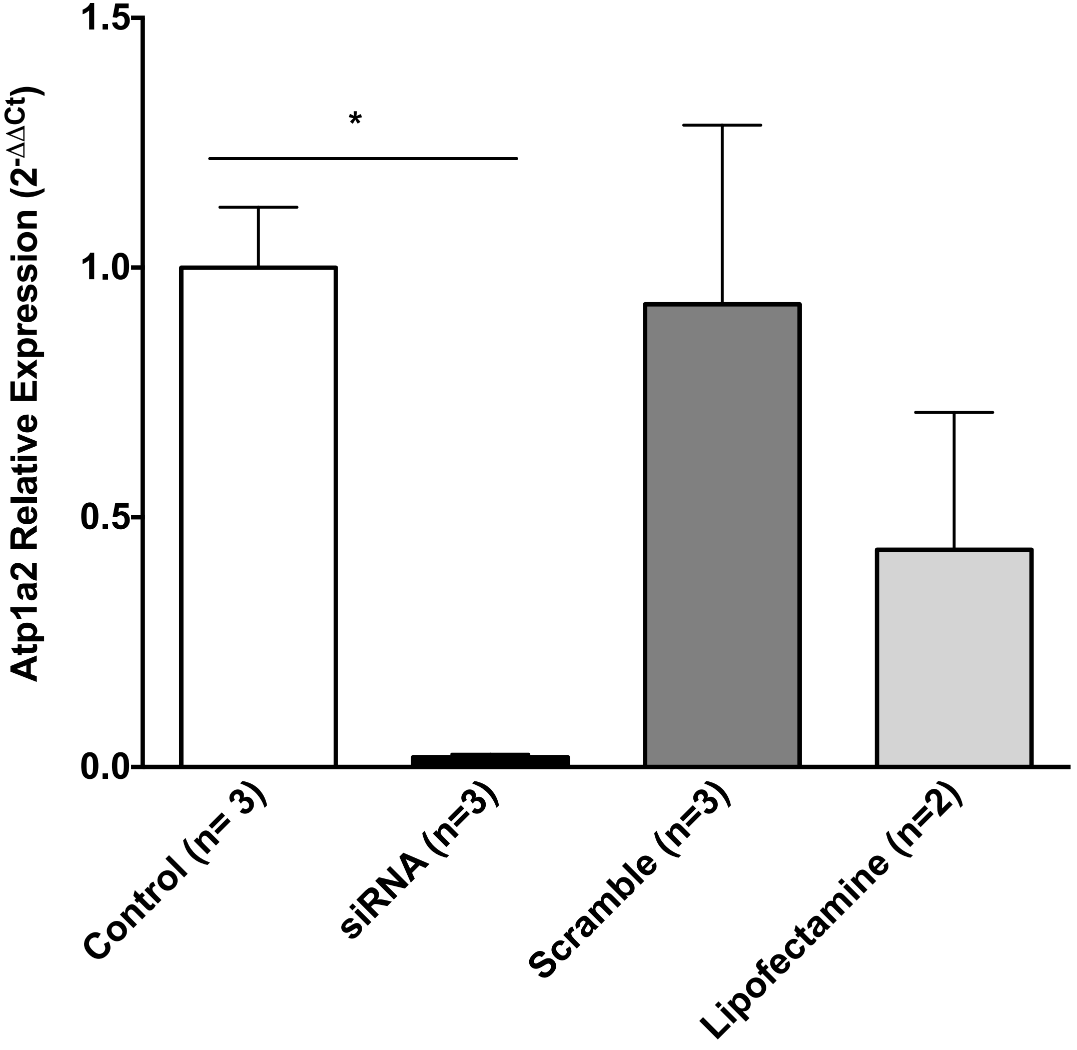

Figure S1. Results from one experiment of qPCR for α2- Na^+,K^+-ATPase. After 48hrs of the silencing process, the RNA was isolated by trizol according to the manufacturer's instructions.The cDNA was prepared and the qPCR was performed in 7500 Fast Real-Time PCR System. Primer sequences (Atp1a2): Forward: AAATCCCCTTCAACTCCACC and Reverse: GATCTCCTTGCCCTGTACC (NCBI Reference Sequence: NM_178405.3).

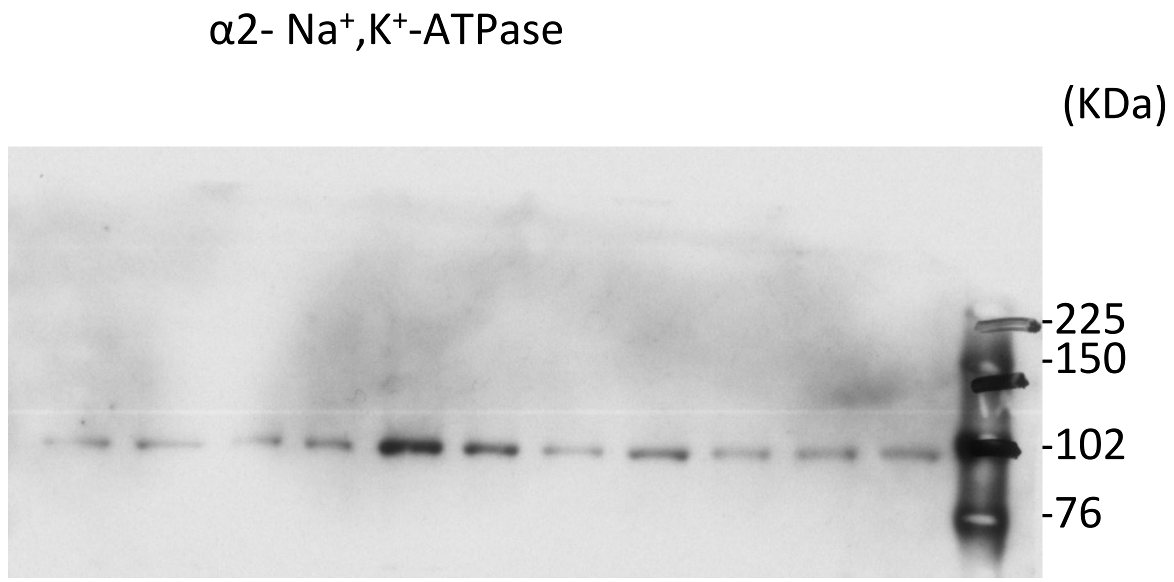

Figure S2. The full-length blot for α2- Na^+,K^+-ATPase for figure 4

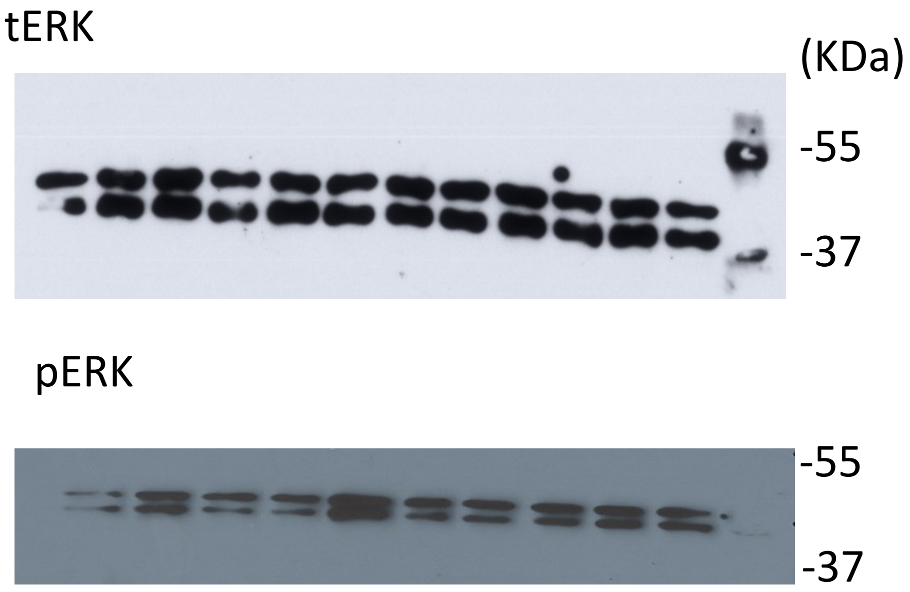

Figure S3. The full-length blots for tERK and pERK for figure 5.

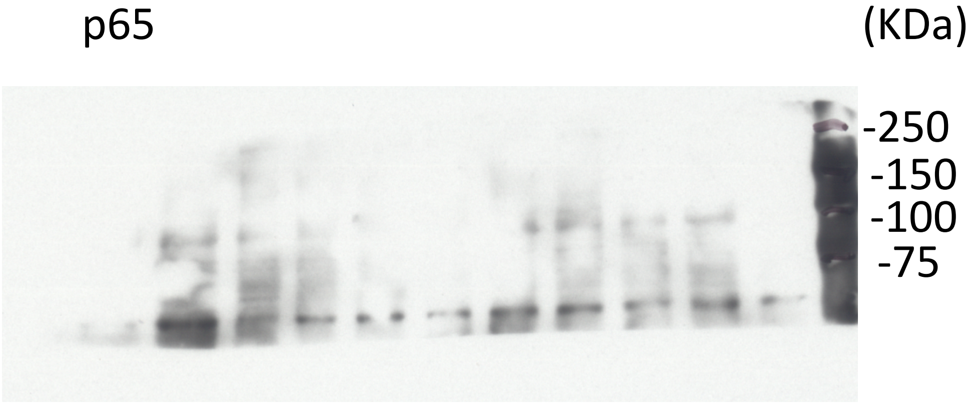

Figure S4. The full-length blots for p65 for figure 5.

A B

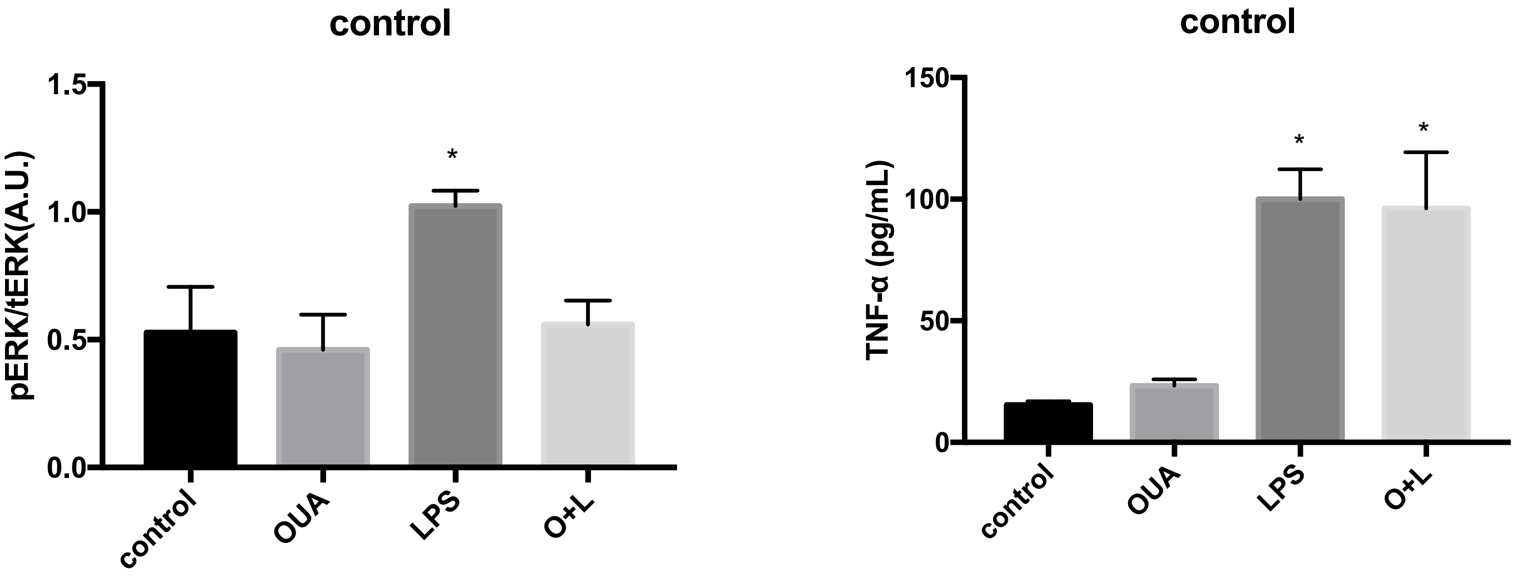

*vs control,OUA and O+L (p<0.05)

*vs control and OUA (p<0.05)

Figure S5. Results from pERK/tERK (A) and TNF-α (B) from mock transfected cells.
